# Filtration efficiency of medical and community face masks using viral and bacterial bioaerosols

**DOI:** 10.1038/s41598-023-34283-9

**Published:** 2023-05-02

**Authors:** Sana Djeghdir, Aurélien Peyron, Gwendoline Sarry, Lara Leclerc, Ghalia Kaouane, Paul O. Verhoeven, Jérémie Pourchez

**Affiliations:** 1 École Nationale Supérieure des Mines de Saint-Etienne, Mines Saint-Etienne, INSERM, U1059 Sainbiose, Centre CIS, Université de Lyon, Université Jean Monnet, 158 Cours Fauriel, CS 62362, 42023 Saint-Etienne Cedex 2, France; 2 GIMAP Team, CIRI, Centre International de Recherche en InfectiologieInserm, U1111, CNRS, UMR5308, ENS Lyon, Université de Lyon, Université Claude Bernard Lyon 1, Lyon, France; 3 Faculty of Medicine, Université Jean Monnet St-Etienne, St-Etienne, France; 4 Department of Infectious Agents and Hygiene, University Hospital of St-Etienne, St-Etienne, France

**Keywords:** Bacteria, Bacteriophages, Biomedical engineering, Disease prevention

## Abstract

Face masks are often recommended in community settings to prevent the airborne transmission of respiratory viruses or bacteria. Our first objective was to develop an experimental bench to assess the viral filtration efficiency (VFE) of a mask with a methodology similar to the normative measurement of bacterial filtration efficiency (BFE) used to determine the filtration performance of medical masks. Then, using three categories of masks of increasing filtration quality (two types of community masks and one type of medical mask), filtration performances measured ranged from 61.4 to 98.8% of BFE and from 65.5 to 99.2% of VFE. A strong correlation (r = 0.983) between bacterial and viral filtration efficiency was observed for all types of masks and for the same droplets size in the 2–3 µm range. This result confirms the relevance of the EN14189:2019 standard using bacterial bioaerosols to evaluate mask filtration, to also extrapolate mask performances whatever their filtration quality against viral bioaerosols. Indeed, it appears that the filtration efficiency of masks (for micrometer droplet sizes and low bioaerosol exposure times) depends mainly on the size of the airborne droplet, rather than on the size of the infectious agent contained in that droplet.

## Introduction

Transmission of aerosol particles is one of the main way for transmission of respiratory infectious agents. It is defined as the passage of pathogenic microorganisms (bacteria or viruses) from a source to a person from infectious aerosols released during exhalatory events generating aerosols, such as breathing, coughing, talking, singing, and sneezing^1^. For example, a single sneeze can release up to 40,000 aerosol particles^2^. From a physical point of view, the term aerosol” corresponds to a heterogeneous mixture of airborne particles, solid or liquid, suspended in a gas and having a relatively low settling velocity^3^ (i.e. typically airborne particles with an aerodynamic diameter lower than 100 µm). However, in the medical literature for decades, a distinction is frequently found, appearing arbitrary (and misleading to an aerosol scientist), between “airborne” particles smaller than 5 µm in diameter and “droplets” larger than 5 µm in diameter^3^. This confusion, emanating from traditional medical language, has sometimes created scientific unfounded terminology distinctions between the so-called "airborne" and "droplet" transmission. Indeed, if people can inhale aerosol particles (of variable size in space and time because they are always dynamic and transitory phenomena), mainly constituted by droplets containing pathogens (from body secretions and excreta), we always breathe liquid airborne particles whatever their size^4^.

Therefore, physical wording, the transmission of respiratory pathogens is done in both cases (“airborne” and “droplets” transmission) by varying sizes of aerosol particles^5^. In other words, whether the pathogen transmission is called "airborne" or "droplet", it can only be by aerosol in all cases. However, it is true that the mode of transmission and control measures may vary according to the physical characteristics of the aerosol particles (including their aerodynamic diameter changing in space and time). On the one hand, if an infectious pathogen is spread mainly by rapidly settling respiratory aerosol particles called “droplets”, the primary transmission control measures consist in reducing direct contact, physical distancing, or the use of face masks. On the other hand, the case of an infectious pathogen whose transmission is mainly called "airborne" requires precautionary measures such as ventilation of the room, air filtration, or attention to the quality and fit of the face mask whenever indoors.

Moreover, it is well recognized that the prevention of infection by airborne pathogens (*e.g.*, influenza, tuberculosis, measles, or coronavirus) can be facilitated through use of mouth-nose cover^3^. Therefore, the use of a face mask is currently recommended to prevent transmission of respiratory diseases for medical staff, contagious patients, and, in some cases, the general population. Obviously, any mask is better than no mask, especially in terms of protecting others. Wearing a mask retains a relatively large proportion of the viral droplets emitted by the mask wearer, thus providing a high degree of protection against bioaerosol emission. Although masks are designed to primarily retain pathogen-laden micrometer-sized aerosol particles when exhaled, they also likely provide some degree of self-protection during inhalation (usually much less due to shrink of liquid aerosol particles between exhalation and inhalation). All things considered, face masks contribute significantly to decrease the risk of infection to those in the vicinity, and may also reduce the risk of infection to the mask wearer, especially if the pathogen is transmitted by larger aerosol particles. For example, the understanding in the nineteenth century on the contagion of tuberculosis caused by the *Mycobacterium tuberculosis* pathogen helped to limit its spread by developing the first mask covering the nose and mouth^6,7^. It was well demonstrated that face masks worn by patients infected with tuberculosis could significantly reduce transmission rates to uninfected patients^8^.

More recently, the global pandemic of coronavirus (COVID-19) has raised the question of the issue of transmission of viral respiratory diseases. At the beginning of the pandemic, the first epidemiological and virological studies favored droplet and surface transmission. Subsequently, numerous studies have shown that transmission by fine aerosol particles containing viable viral particles represents one of the main routes of transmission of severe acute respiratory syndrome Coronavirus 2 (SARS-CoV-2) in poorly ventilated indoor environments^1,9,10^. Early on, given the knowledge of airborne diseases, the Centers for Disease Control and Prevention (CDC) and the World Health Organization (WHO) advocated universal mask use to reduce the risk of SARS-CoV-2 transmission^11^. Masks were indicated to prevent others by limiting the exhalation of potentially infectious respiratory droplets containing SARS-CoV-2 into the air stream, but also to protect the wearer in many cases^3^. The emergence of COVID-19 has thus confirmed the mask effectiveness. Indeed, accumulated evidence shows that face masks are a critical barrier, reducing the number of infectious viruses in the exhaled air, especially in asymptomatic or pre-asymptomatic individuals^12–14^. A study by Bagheri et al. (2021) showed that if an uninfected person wears a surgical mask and an infected person speaks without a mask, the maximum risk achieved in this case is 90% after 30 min for the uninfected person. However, if both people wear surgical masks, the maximum risk is less than 30% even after one hour^15^.

Among the different categories of face masks, medical face masks (MFMs) are single-use medical devices specially designed to prevent the dissemination of bioaerosols from the wearer into the environment. They are regulated by specific standards such as the European standard EN14683:2019^16^ and must therefore meet performance requirements, particularly in terms of bacterial filtration efficiency (BFE). MFMs are classified into two types according to their BFE values. Type I masks (BFE ≥ 95%) and Type II/IIR masks (BFE ≥ 98%). By contrast, community face masks (CFMs) or cloth masks come in various designs and are made with a large variety of fabrics and whilst not as effective as MFMs. CFMs are not designed to be used in an environment requiring high level of sanitary protection. Numerous studies performed on CFMs have shown that various characteristics of the fabrics (material type, fabric type (woven or knitted), fiber characteristics) may influence their filtration^17,18^. CFMs can be considered as simply anti-spray masks and are typically reused by washing. Unlike the MFMs that are strictly regulated and certified, CFMs are not standardized nor strictly regulated. Currently CFMs are intended for the general population and divided into two categories according to their ability to filter particles of a size of 3 ± 0.5 μm. According to AFNOR SPEC S76-001 requirement^19^, the CFMs of category 1 must have a filtration efficiency higher than 90%, whereas the CFMs of category 2 must have a filtration efficiency higher than 70%.

Mask filtration is based on different mechanisms: gravity sedimentation, inertial impaction, interception, diffusion and electrostatic attraction^20^. Due to the significant effect of many factors on the performance of mask filtering (and mainly the type of mask, CFMs *vs.* MFMs for instance), clarifying the mechanism of bioaerosol penetration into the mask has high importance. The first and dominant mechanism is the filtration of pathogen-laden droplets directly through the filter material. This first step is mainly dependent on the size and speed of the airborne droplet for a given filter material design. But when contaminated aerosol liquid particles reach the outer surface of the mask, if the surface does not destroy the pathogen initially contained on it, microorganisms can then penetrate the mask during breathing by various mechanisms (including capillaries)^20^. This second step is mainly dependent on the size and the number of pathogens accumulated at the external surface of the mask if the exposure time is long enough for a given filter material design. Thus, a mask can often become a pathogen collector, particularly when its outer surface is exposed to contaminated aerosol particles. Since viruses and bacteria can remain on the surface of the mask, or even in the textile structure of the masks, it is obviously dangerous and undesirable that they can migrate through the mask once the filtration of the liquid aerosol particles by the filtering material has been achieved.

The determination of the penetration and propagation capacities of micro-organisms through the mask therefore appears to be a major challenge for evaluating the protection provided by the mask. It is essential to analyze the filtration of bioaerosols via the prism of the transport of pathogens contained in the airborne liquid vectors through the mask, rather than stopping only at the study of the filtration of these aerosol particles at the surface of the filtering material. This work was carried out in order to study, for a fixed size of aerosol liquid particles (in the range of aerodynamic diameter between 2 and 3 µm), and for different qualities of mask (a MFM, a CFM with excellent performances, and a CFM with a low filtration efficiency), the impact of the size of the pathogen (a virus of 100 nm or a bacterium of 1 µm) on the penetration through the mask. In other words, since MFMs are evaluated using bacterial and non-viral bioaerosols, this study allows us to study whether an extrapolation (for a fixed aerosolized vector size of a few microns of aerodynamic diameter) can easily be carried out between Bacterial Filtration Efficiency (BFE) and Viral Filtration Efficiency (VFE).

## Materials and methods

### Face masks

In this study, three types of masks were tested: 2 CFMs (Oriol & Fontanel company, CFM type 1, France; CJ Textile company, CFM type 2, France), and one MFM (Bioserenity company, type IIR, France) (Table 1). Measurements were performed on masks samples of a minimum size of 100 mm by 100 mm, including all layers. In accordance with the EN14683:2019 standard, the masks were preconditioned at 21 ± 5 °C and 85 ± 5% relative humidity for 4 h to reach atmospheric equilibrium before testing. Experiments were performed on at least five samples for each mask type with the inside of the mask in contact with airborne pathogens.

**Table 1:** Masks tested in this study.

Type of mask	Medical face mask type IIR (MFM)	Community face mask type I (CFM1)	Community face mask type II (CFM2)
Main features of the mask structure	3 layers of non-woven polypropylene fibers (spunbound, meltblown, and spunbound layers) Pore size of the spunbound layer ≈ 100 µm Pore size of the meltblown layer ≈ 20 µm Fiber diameter of the spunbound layer = 25 ± 1 µm Fiber diameter of the meltblown layer = 6 ± 3 µm	CFM is made up 2 different layers Pore size of the inner layer ≈ 300 µm Pore size of the outer layer ≈ 50 µm Fiber diameter of the inner layer = 31 ± 5 µm Fiber diameter of the outer layer = 18 ± 1 µm	CFM is made up 2 identical layers Pore size ≈ 200 µm Fiber diameter = 18 ± 0 µm
Electron microscopy images of the microscopic structure Scale bars correspond to 30 µm (MFM) or 10 µm (CFM) 400× magnification			
Optical microscopy images of the microscopic structure Scale bars correspond to 300 µm 4× magnification			
Expected conformity in term of filtration efficiency from the manufacturer	> 98% of BFE	> 90% of PFE	> 70% of PFE
	EN14683:2019 standard	AFNOR SPEC S76-001 requirement	

*PFE* particle filtration efficiency, *BFE* Bacterial filtration efficiency.

The microscopy analyses were made using a Leica DM LB Microscope with a C Plan lens model. The images were taken with a Bresser MikroCam SP 5.0 at 4× magnification. Scanning Electron Microscopy (SEM) was performed on surfaces of the masks using a JEOL JSM-6500F. Samples were mounted on brass support with double sided carbon tape and coated with 14 nm of gold (Quorom Q 150R ES). Images were taken with beam accelerating voltage of 5 keV. For non-woven materials (such as meltblown of MFM), fibers are randomly oriented. By contrast, for woven and knitted materials (such as layers of CFM) contain yarns (bundles of fibers) that are interlaced to each other. The pores are formed at yarn interstices for the woven and knitted fabrics whilst they are formed by small spaces between individual fibers in non-woven filters. The spaces in between yarns were considered as the pores for the community face masks. Although the pore shape and size in community face masks were not uniform, we tried to extract quantitative information on the size of the inter-yarn pores by measuring the longest dimension of each inter-yarn pore. These measurements provided an estimation of the size of an inter-yarn pore in each community face mask (Table 1).

### Bacterial filtration efficiency (BFE)

**Figure 1:**
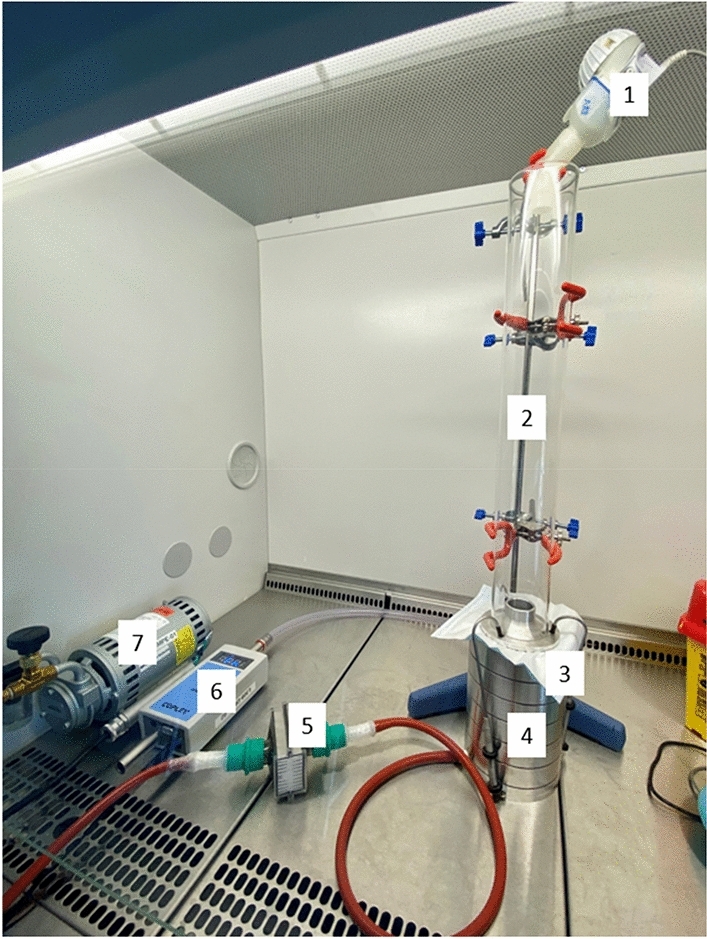
The BFE experimental set-up according to EN14683:2019 standard. (1) Nebulizer, (2) aerosol chamber, (3) sampling materiel, (4) cascade impactor, (5) filter, (6) flow meter, (7) vacuum pump.

The evaluation of the BFE was performed according to the EN14683:2019 standard (6) using an experimental procedure previously developed and then validated^17,21,22^. The test bench used was in conformity with the specifications of the standard with minor adaptations (e.g. size of the aerosol chamber) which did not prevent us from obtaining an accreditation by an external organism proving the validity of the measurements for obtaining the CE marking. The experimental set-up is illustrated in Fig. 1. Briefly, an aerosol stream containing a known load of *Staphylococcus aureus* (ATCC 6538 at 10^6^ UFC/mL before dilution) is generated using a vibrating mesh nebulizer (E-flow, PARI GmbH, Starnberg, Germany). Then the bioaerosol drawn through a glass aerosol chamber (445 mm long and 60 mm outer diameter) using a vacuum pump at a constant flow rate of 28.3 L min^−1^. Mask samples are attached between the aerosolization chamber and a viable six-stage Andersen Cascade Impactor (ACI, Tisch environmental, Miami, USA). The ACI led to collect and classify the bioaerosol into 6 size fractions (ranging from 7 µm for the first stage to 0.65 µm for the sixth stage) according to their inertia and consequently their aerodynamic diameter. A 90 mm plastic Petri dish, containing agar culture medium, used as an impinger plate is placed at each ACI stage to collect the airborne bacteria for each aerodynamic size fraction. The EN14683:2019 standard imposes two main specifications for the BFE normative procedure: (i) a mean number of CFU for all ACI stages between 1700 and 3000 CFU, (ii) a mean particle size (MPS) of 3.0 ± 0.3 µm. Obviously, these two aerosol specifications (MPS and total CFU) can only be valid for positive control runs (because in the presence of a mask, if the filtration is very efficient, it is possible and frequent that no or very few bacteria pass through the mask and reach the cascade impactor to measure MPS and total CFU). The MPS parameter is calculated using the 50% effective cut-off diameters following Eq. (1).

1 $$MPS= \frac{\left(P1\times C1\right)+\left(P2\times C2\right)+\left(P3\times C3\right)+\left(P4\times C4\right)+\left(P5\times C5\right)+(P6\times C6)}{C1+C2+C3+C4+C5+C6}$$

Mean particle size (MPS) calculation. (Px, x = [1–6]) is the particle diameters corresponding to a 50% sampling efficiency of each of the six stages and (Cx, x = [1–6]) is the number of viable particles obtained from each of the six petri dishes.

A measurement cycle for one type of mask includes eight successive tests. At first, a positive control is performed without the presence of a mask placed between the ACI and the aerosol chamber. This positive control was used to determine the number of viable particles used in each test (and thus to verify that the specification required by the EN14683:2019 standard is within the range of 1700–3000 CFU). Next, five experiments are performed to measure the filtration efficiency of the masks (i.e., test samples), changing the mask for each experiment at the inlet of the ACI cascade impactor. Then, a second positive control experiment is performed. Finally, this cycle of eight consecutive experiments ends with a negative control consisting of passing air, without adding pathogens, for 2 min (this serves as a contamination check to verify that bacteria/viruses were deposited during the positive run and that the test samples were only from the bioaerosol source). The petri dishes, capturating the bacteria at each stage of the ACI for the eight experiments, were incubated at 37 ± 2 °C for 22 ± 2 h. The CFU were counted with an automatic colony counter Scan 4000 (Interscience, Saint Nom la Bretèche, France).

### Viral filtration efficiency (VFE)

VFE test is not a standardized test method, but was adapted from the BFE test described in the EN14683:2019 standard (and from the experimental methodology in “Bacterial filtration efficiency (BFE)”). The filtration efficiency was measured using phi11 bacteriophage (Lab stock from Centre International de Recherche en Infectiologie, GIMAP team, Université de Lyon, Inserm) instead of *Staphylococcus aureus* in the BFE test. A suspension in PBS solution of phi11 at 10^8^ PFU/mL (PFU for plaque-forming unit) was aerosolized using a vibrating mesh nebulizer (E-flow, PARI GmbH). The same experimental bench used for the BFE test is then used to perform the VFE test as illustrated in Fig. 1. Briefly, the viral bioaerosol drawn through a glass aerosol chamber and an ACI using a vacuum pump, and the mask samples are attached between the aerosolization chamber and the ACI. Aerosolized bacteriophages were captured in Phospate-Buffered Saline (21-040-CV, Corning, Manassas, USA) filled petri dishes placed in the ACI stages (15 mL of PBS per petri dish). An assessment of the viability of the collected viruses (not neutralized) at each stage of the ACI is performed by counting the lysis patches on Columbia blood agar plates flooded with *Staphylococcus aureus* RN4220 (bacterial strain sensitive to phi11 bacteriophage). The plates are then incubated at 37 °C for 22 ± 2 h. The PFU represents the number of particles or droplets of viral aerosol. The measurement cycle for each mask type and the calculation of the MPS were performed as described for the BFE method. The PFU were counted manually.

### Filtration efficiency calculation

The filtration efficiency (FE) of the mask, expressed as a percentage, is calculated following Eq. (2). The FE parameter was determined by measuring the number of CFU for the BFE test (respectively the number of PFU for the VFE test), passing through the mask material compared to a positive control with no filter material was placed at the inlet of the ACI.

2 $$FE= 100 \times \frac{C-T}{C}$$

where FE is the filtration efficiency, C is the mean of the two positive runs of the total of six plate counts, and T is the total of the six plate counts for each test sample.

### Statistical analysis

Statistical analyses were performed with GraphPad Prism 9.4.1 (GraphPad Software, San Diego, CA, USA). A two-way Anova with Sidak post-hoc test was used to assess whether there were significant differences between the filtration efficiency results of the BFE test method and the VFE test method. P-values < 0.05 were considered significant. Correlation coefficients for the comparison of filtration efficiency between the test methods were obtained using Excel.

## Results and discussion

Table 2 shows the filtration efficiency for bacteria and virus loaded droplets of similar aerodynamic size (i.e. in the range 2–3 µm), and their standard deviations for the three types of mask quality (MFM, CFM1 and CFM2). First, we observe that the mean MPS for the BFE test is slightly higher than the mean MPS obtained for the VFE test (2.9 ± 0.1 µm versus 1.9 ± 0.2 µm). Thus, a change in MPS is reported between the BFE and VFE experiments, although the same test rig was used. The possible source of this change in MPS may be induced by a slight change in the physical characteristics of the solution used to suspend the virus (for the VFE assay) or bacteria (for the BFE assay) before being aerosolized. Indeed, it is well known that the characteristics of the solution (e.g. viscosity or protein concentration) play a key role in the aerosol generation process and the fate of the aerosol particles.

**Table 2:** Bacterial filtration efficiency (BFE) and viral filtration efficiency (VFE) values (expressed in % as defined in Eq. (2) at Filtration efficiency calculation) with respective mean particle size (MPS in µm) for the different types of masks evaluated in this study (MFM, CFM1 and CFM2; MFM refers to medical face mask, CFM refers to community face mask).

Mask	Bacterial filtration			Viral filtration		
	BFE (%)	MPS (µm)	Total CFU for positive control runs	VFE (%)	MPS (µm)	Total PFU for positive control runs
MFM	98.8 ± 0.2	2.92 ± 0.1	2057 ± 264	99.2 ± 1.0	2.14 ± 0.2	2931 ± 756
CFM1	89.7 ± 3.6	2.96 ± 0.1	2576 ± 57	85.7 ± 4.7	1.94 ± 0.1	3719 ± 241
CFM2	61.4 ± 1.2	2.76 ± 0.1	2197 ± 356	65.5 ± 8.0	1.74 ± 0.6	2390 ± 559

**Figure 2:**
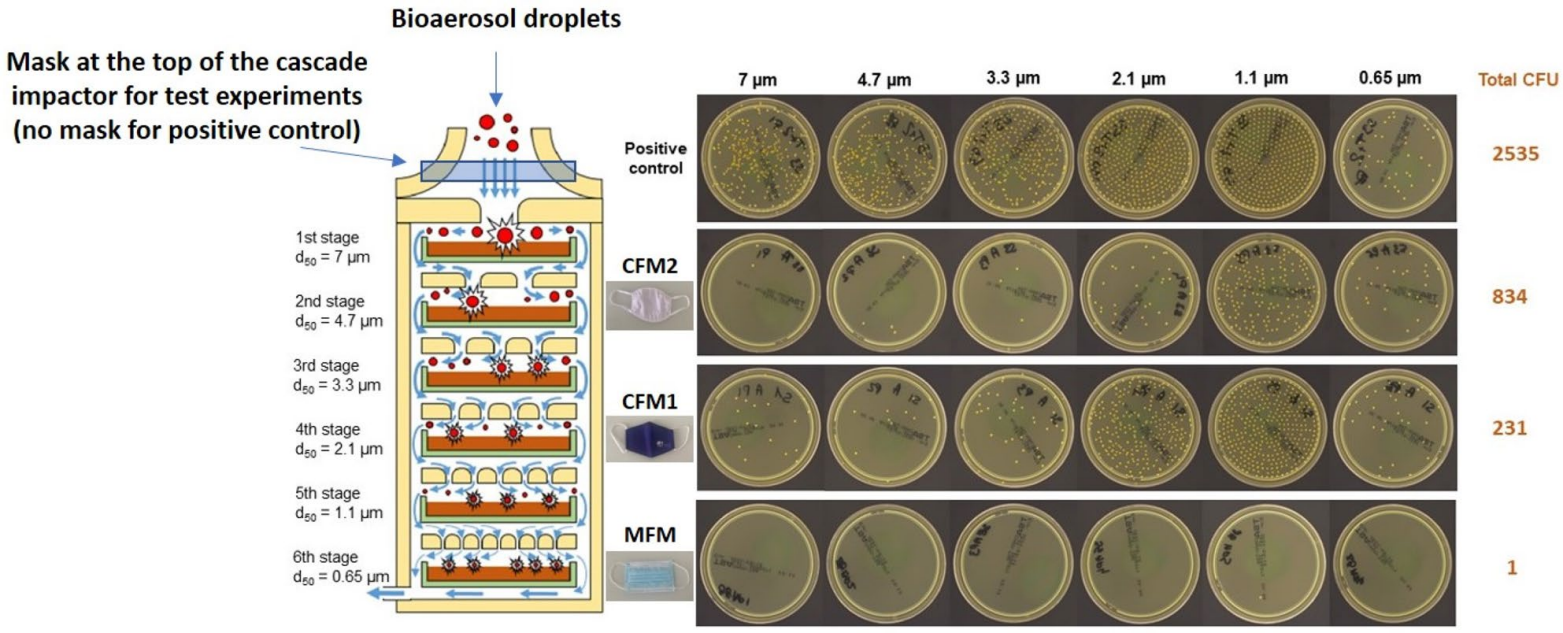
(Left) Schematic of a six-stage viable ACI (yellow). Airborne particles (red) are aspirated inside the cascad e impactor with a 28.3 L/min flow rate (blue arrow). Each stage of the ACI contains a Petri dish (green) filled with nutrient agar (brown). (Right) Images of a BFE test for each impaction stage for the three mask types (MFM, CFM1 and CFM2; MFM refers to medical face mask, CFM refers to community face mask). d50 refers to the cut-off size of the cascade impactor stage. The colonies on the Petri dishes were observed using an HD colony counter.

**Figure 3:**
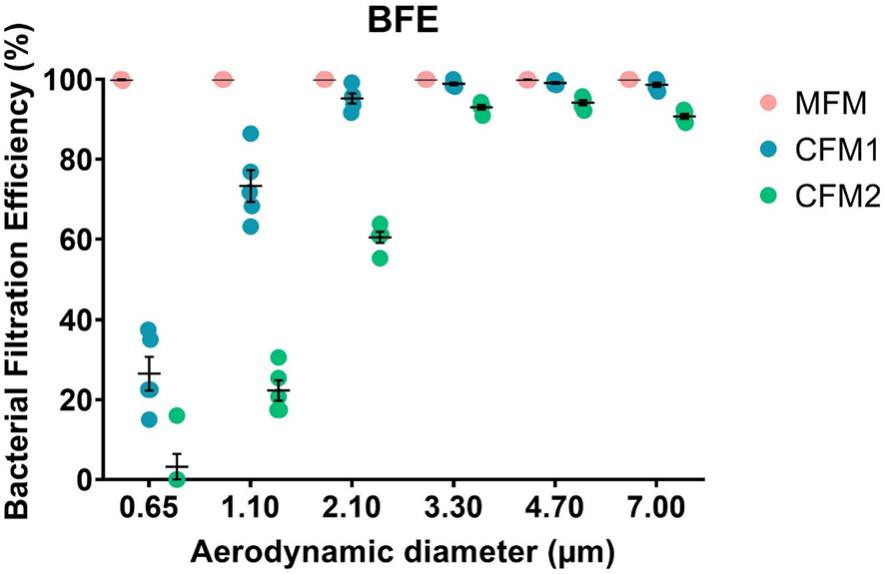
Spectral bacterial filtration efficiency (expressed in %) as a function of aerodynamic particle diameter (expressed in µm) for the three types of mask (MFM, CFM1, CFM2; MFM refers to medical face mask and CFM refers to community face mask); experimental values (N = 5), mean with standard deviation.

**Figure 4:**
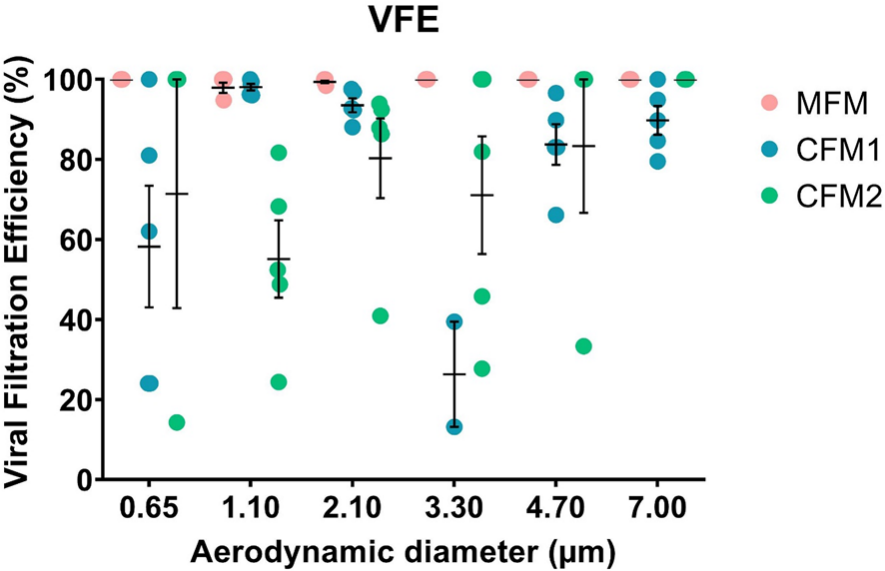
Spectral viral filtration efficiency (expressed in %) as a function of aerodynamic particle diameter (expressed in µm) for the three types of mask (MFM, CFM1, CFM2; MFM refers to medical face mask and CFM refers to community face mask); experimental values.

Despite this difference of approximately 1 µm for the MPS (i.e. the size of the microorganism-laden droplets) for the BFE and VFE tests, we support the conclusion that the aerodynamic properties of the droplets are very similar in this 2–3 µm range in terms of the filtration efficiency of the mask (in other words, this slight difference in droplet size has no impact on the resulting filtration efficiency). This is strictly the case when the vector size distribution peaks between 2 and 3 microns. But having an MPS in the 2–3 micron range does not guarantee that the vector size distribution is concentrated at that size. Nevertheless, the data we have already published on the size distribution of bioaerosols generated by our experimental bench for these tests, confirm that our hypothesis is legitimate. Indeed this is a reasonable assumption based on our experience with spectral mask filtration using the BFE test^18^. Moreover, spectral filtration efficiency obtained with the BFE test (Figs. 2 and 3) clearly demonstrate that comparable and high bacterial filtration performance is observed for MFM and CFM1 for vector ranges between 2–3 µm. Doubtless, the CMF2 shows a significant reduction in bacterial filtration efficiency from a particle size of 2.1 µm (Figs. 2 and 3). These results confirm our previous hypothesis that the slight difference in droplet size between the BFE and VFE tests, with MPS remaining overall in the 2–3 µm range for both tests, does not have a significant impact on mask filtration efficiency (Figs. 2 and3). Besides, quite similar conclusions are observed for the spectral filtration efficiency obtained with the VFE test (Fig. 4), with overall excellent filtration efficiency whatever the vector size for MFM and a filtration efficiency that decreases with vector size for CFM1 and CFM2 (although the difference in filtration performance between CFM1 and CFM2 is less readable in the case of the VFE measurements compared to the BFE measurements, due to a larger standard deviation of the measurement).

Regarding the BFE results (Table 2), medical masks show values above 98% (in good accordance with Type IIR specification according to EN14683:2019). The community masks present a lower bacterial filtration efficiency compared to MFM equals to 89.7 ± 3.6% for the CFM1, and 61.4 ± 1.2% for the CFM2. We can notice that the bacterial filtration efficiency obtained for both types of community masks is in relatively good compliance with the PFE limit of 90% for CFM type 1 and of 70% for CFM type 2.

**Figure 5:**
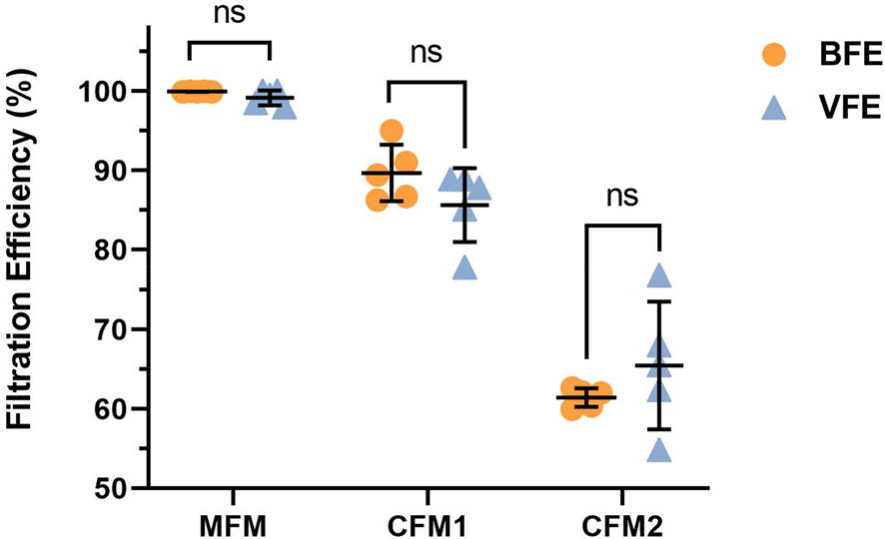
Filtration efficiency values (expressed in %, as defined in Eq. 2 at "Filtration efficiency calculation”) between bacterial filtration efficiency (BFE) and viral filtration efficiency (VFE) methods for the different types of masks evaluated in this study (MFM, CFM1 and CFM2; MFM refers to medical face mask, CFM refers to community face mask). *ns* not significantly different; experimental values (N = 5), mean with standard deviation.

Concerning the VFE test, the results clearly demonstrate a good correlation between bacterial and viral filtration efficiency for the different qualities of masks tested. By comparing the results of the two methods (Fig. 5), we can conclude that the VFE and BFE tests converge towards similar values whatever the mask quality (MFM, CFM type 1 or CFM type 2). Indeed, the statistical comparison of the two tests showed no significant difference (p > 0.05). The BFE values were undoubtedly correlated to the VFE values since the results (Supplementary Fig. S1) showed a good correlation (r = 0.983) between the two methods for a similar range of aerodynamic droplet size in the 2–3 µm range. However, we should be cautious considering the very limited number of points in the correlation plot (n = 3) statistical significance of this finding is limited.

**Figure 6:**
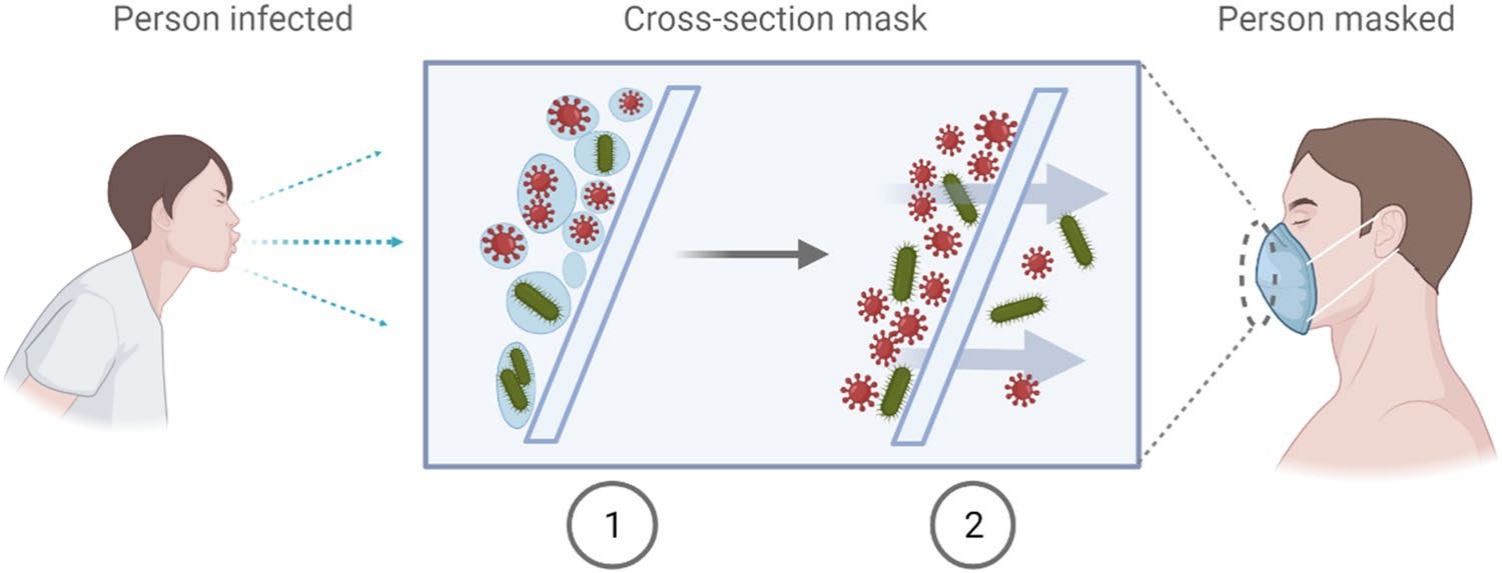
Mechanisms of the aerosols penetration. (1) Filtration of pathogen-laden droplets directly through the filter material. (2) Migration through the filter material of pathogens accumulated on the surface of the mask.

The filtration of aerosol droplets through a face mask is governed by two main mechanisms (Fig. 6). The first mechanism is the direct filtration of micrometer-sized droplets loaded with pathogens by the filter material of the mask. This first step depends mainly on the size of the transmission vector (i.e. droplets of the order of 2–3 µm in our case). The second mechanism consists in the transport of the pathogen, by air or after deposition of the droplets on the mask surface, through the mask. This second step depends mainly on the size and number of pathogens (micrometric size for bacteria, 100 nm for viruses) accumulated on the external surface and in the textile structure of the mask. Indeed, the accumulation of pathogens on the mask can lead to their penetration through the mask for a sufficiently long exposure time. The higher the respiratory rate of the exposed person, the greater the penetration of micro-organisms.

The results show that the first filtration mechanism is undoubtedly predominant on the filtration efficiency of a mask, whatever its quality (MFM, CFM type 1 or type 2). Indeed, we demonstrated that similar filtration efficiencies are obtained for the same range of droplets (in the 2–3 µm range) and this whatever the size of the pathogen used (bacteria versus virus). Thus, from these original data, we can conclude that the filtration efficiency of the mask (whatever the quality of the mask) depends mainly on the size of the vector containing the pathogen, and not on the size of the pathogen itself. Pragmatically, these results also confirm that the use of EN14683:2019 (using bacterial bioaerosols) to evaluate the performance of medical masks can be extrapolated with confidence in terms of mask filtration efficiency against viral bioaerosols (for the same size of aerosolized vector in the range of about 2 to 3 µm). Furthermore, in addition to aerosol filtration/penetration mechanisms, to properly assess the protection factor of a face mask, the literature has also shown that other factors are important, such as in particular the leaking fraction for loose fitting masks such as those presented in this study.

However, caution should also be exercised because the EN14683:2019 regulatory test is performed on short exposure times (1 min of exposure of the mask to the bioaerosol during nebulization, followed by 1 min of contact between pathogens on the mask surface with a flow rate of 28.3 L/min). As noted earlier, exposure time is a very important parameter for the second filtration mechanism described in Fig. 5. Thus, it is not impossible that the design of the EN14683:2019 regulatory test primarily favors the evaluation of the first filtration mechanism (filtration of pathogen-laden droplets) compared to the second filtration mechanism (pathogen transport through the mask). Future studies, modifying the conditions imposed by the EN14683:2019 regulatory framework, could be undertaken to evaluate bacterial or viral filtration efficiency over longer exposure times (i.e., longer than the 2 min imposed by EN14683: 2019), in order to further examine the potential impact of the second filtration mechanism on filtration efficiency of face masks under experimental conditions that would possibly be more favorable to it (higher pathogen saturation of the external surface of the mask by the microorganisms, higher duration to allow more pathogen transport through the mask).

For many epidemiologists as well as health agencies and the WHO, the 5 µm threshold is used to distinguish between airborne and droplet transmission. Droplet transmission would be caused by particles larger than 5 µm in diameter, while only particles smaller than 5 µm would allow airborne transmission. But from a physical point of view, this value of 5 µm is mainly arbitrary and has no particular meaning. Thus, there is no "true" aerodynamic size boundary between these two modes of pathogen delivery (airborne vs. droplet transmission).

The VFE is a non-regulatory test for evaluating mask filtration which in our study gave values quite similar (for droplet ranges in the 2–3 µm range) to the standard BFE value according to EN14683:2019. The results of our research are supported by other studies. Whiley et al. (2020) showed that the VFE values of surgical masks (VFE = 98.5% against aerosols with an average size of 2.6 µm) were comparable to their manufacturer's advertised BFE. We thus confirm that the VFE test is no more interesting than the BFE test when it comes to micrometric droplet ranges as in our study. On the other hand, it can be interesting to use the VFE test to assess the performance of masks against airborne transmission of pathogens (i.e. for airborne droplets typically smaller in the submicron range). Indeed, it is simply impossible to measure the performance of filtration efficiency against submicron bioaerosols with a BFE test, because the size of bacteria is about 1 micron. The BFE test is therefore perfectly suitable for testing the efficiency of filtration masks against airborne micrometric droplets (corresponding rather to the “droplet transmission”), but can never be adapted to assess performance in the case of airborne submicrometric droplets (corresponding rather to the “airborne transmission”). A VFE is therefore the only test that could really evaluate the quality of a mask against submicron size vectors. It would be possible to create a VFE test by generating viral bioaerosols consisting in airborne virus-laden droplets smaller than 1 µm using specific nebulizers such as the one we have already characterized in a previous study and presenting an aerodynamic diameter distribution of 0.15 to 0.5 µm.

## Conclusion

The bacterial filtration efficiency (BFE) and viral filtration efficiency (VFE) of medical and community face masks were compared. The results showed a very good correlation between the two filtration methods (viral *versus* bacterial) for a droplet size range of 2–3 µm. This result confirms the relevance of using the results of the EN14189:2019 standard, using bacterial bioaerosols, to assess the filtration performance of medical masks against bioaerosols of viral microorganisms for micrometric droplet sizes. In other words, the EN14189:2019 normative procedure appears rather suitable for assessing the filtration of masks against micron droplets (the case of the so-called “droplet transmission”), whatever the pathogen (bacteria or virus). Indeed, it appears that the filtration efficiency of the masks (for the size of droplets of the order of 2–3 μm and for the operating conditions set by these normative procedures and in particular for low exposure durations to bioaerosols) depends mainly on the size of the airborne droplet rather than the size of the infectious agent contained in this droplet.

## Supplementary Information

Supplementary Figure S1.

## Data Availability

All main data are available in the main text or the supplementary materials. Complementary information upon data used in the analysis are available on reasonable request to corresponding author.
