# Supplementary material for: Filtration efficiency of medical and community face masks using viral and bacterial bioaerosols
Source: Sci Rep. 2023 May 2;13:7115. doi: 10.1038/s41598-023-34283-9 (PMC10152419; doi:10.1038/s41598-023-34283-9)
Supplement: Supplementary file 1 — Supplementary Figure S1. [file 41598_2023_34283_MOESM1_ESM.docx]

**Supplementary Figure S1:** Correlation between viral and bacterial filtration efficiency. Pearson correlation coefficient (r) equals to 0.983.
